# Dimensional Accuracy of a Sterilized and Disinfected 3D-Printed Surgical Guide: An In Vitro Study

**DOI:** 10.3390/microorganisms13112457

**Published:** 2025-10-27

**Authors:** Sultan Meteb Alshammari, Abdulrahman Jafar Alhaddad, Thamer Y. Marghalani, Walaa A. Babeer, Samar Hatem Abuzinadah

**Affiliations:** 1 Oral and Maxillofacial Prosthodontic Department, Faculty of Dentistry, King Abdulaziz University, Jeddah 21589, Saudi Arabia; aalhaddad@kau.edu.sa (A.J.A.); tmarghalani@kau.edu.sa (T.Y.M.); wababeer@kau.edu.sa (W.A.B.); 2 Restorative and Digital Dentistry, Restorative Dentistry Department, Faculty of Dentistry, King Abdulaziz University, Jeddah 21589, Saudi Arabia; sabuzinadah1@kau.edu.sa

**Keywords:** surgical guide, 3D printing, surgical guide accuracy, sterilization, disinfection

## Abstract

Despite the widespread use of surgical guides, there is no universal sterilization protocol. Surgical guides are often designed for single use, but can become contaminated, which increases the risks of infection and implant failure. This study evaluates the effects of alcohol immersion, alcohol spray, low temperature, and steam sterilization on the dimensional stability of surgical guides to ensure accurate implant placement and reduce failure. One standard dental model was scanned using a laboratory scanner. Ninety guides were printed and were then divided into six groups allocated as control, alcohol spray, alcohol immersion with ultrasonication, low-temperature dry sterilization, and two autoclave methods. Specimens were stored in dry–dark media and scanned at 0, 3, and 7 days, with dimensional changes assessed using CloudCompare. The Shapiro–Wilk, Levene’s, Repeated measures one-way ANOVA, and Tukey’s post hoc tests were used to determine statistical differences. Time significantly affects stability, with RMS values improving over time. Autoclave 121 °C and low-temperature 54 °C sterilization showed the lowest RMS values, indicating better stability. Within the limitations of the present study, the most effective approach for maintaining the dimensional stability of surgical guides was autoclaving at 121 °C, +1 bar for 20 min, and the second-best technique was low-temperature dry sterilization at 54 °C for 1 h.

## 1. Introduction

Modern three-dimensional (3D) imaging and virtual planning tools have been effectively utilized in the maxillofacial region for the pre-operative planning of surgical procedures. When combined with 3D printing or medical additive manufacturing (AM), these innovations facilitate the integration of virtual planning at the surgical site through patient-specific surgical guides [1].

It is crucial to consider anatomical, biological, and esthetic variables during planning and implant placement to avoid potential biological and technical complications [2,3].

One of the primary biological concerns is the risk of infection at the operative site, which can compromise the successful osseointegration of the implant, as reported by Camp-Font et al. Only 33.5% of implants affected by surgical site infection can ultimately be retained. Moreover, antibiotic therapy alone proves to be ineffective in 89% of surgical site infection cases, where reevaluation of the surgical site is necessary to manage the infection adequately [4].

In fact, surgical guides are mostly used in flapless procedures; however, unexpected complications may sometimes necessitate flap reflection for better visualization and management. Despite the widespread usage of surgical guides, there is no universally accepted best practice for 3D-printed surgical guide sterilization [5,6].

Although they are individually manufactured in a dental laboratory for single use, the surgical guides can become contaminated during production. Common microorganisms include Pseudomonas Aeruginosa, Acinetobacter baumannii, Enterococcus faecalis, Enterococcus faecium, Staphylococcus aureus, Enterobacter cloacae, Escherichia coli, and Candida Albicans. When these guides are used in surgical procedures, they come into contact with open wound areas, exposed bone, and the bloodstream, increasing the risk of infection [2].

Although many commonly used 3D printing materials for surgical guides are not currently considered sterilizable. This lack of standardization presents additional challenges to maintaining effective infection control [2].

The biological benefits of autoclaving surgical guides outweigh the potential risks of alteration in flexural properties or Vickers hardness [7]. A limited number of recent studies have measured the distortion of surgical guides and their consequent impact on implant placement accuracy caused by steam sterilization. Several of these investigations identified statistically significant changes in linear dimensions [1,8]. However, statistical significance does not always equate to clinical relevance regarding implant placement accuracy, as no significant volumetric change was observed [9]. Precise implant positioning [10] and maintaining an aseptic surgical field are crucial for clinicians to achieve successful implant interventions [11].

## 2. Objectives

This study aims to evaluate the impact of alcohol immersion, alcohol spray, low temperature, and steam sterilization on the dimensional stability of surgical guides, ensuring accurate implant placement and reducing the incidence of implant failure due to infection. The null hypothesis of this study is that there is no statistically significant difference in the dimensional stability of surgical guides after sterilization, low temperature, alcohol spray, or immersion at 3 to 7 day intervals.

## 3. Materials and Methods

**Figure 1:**
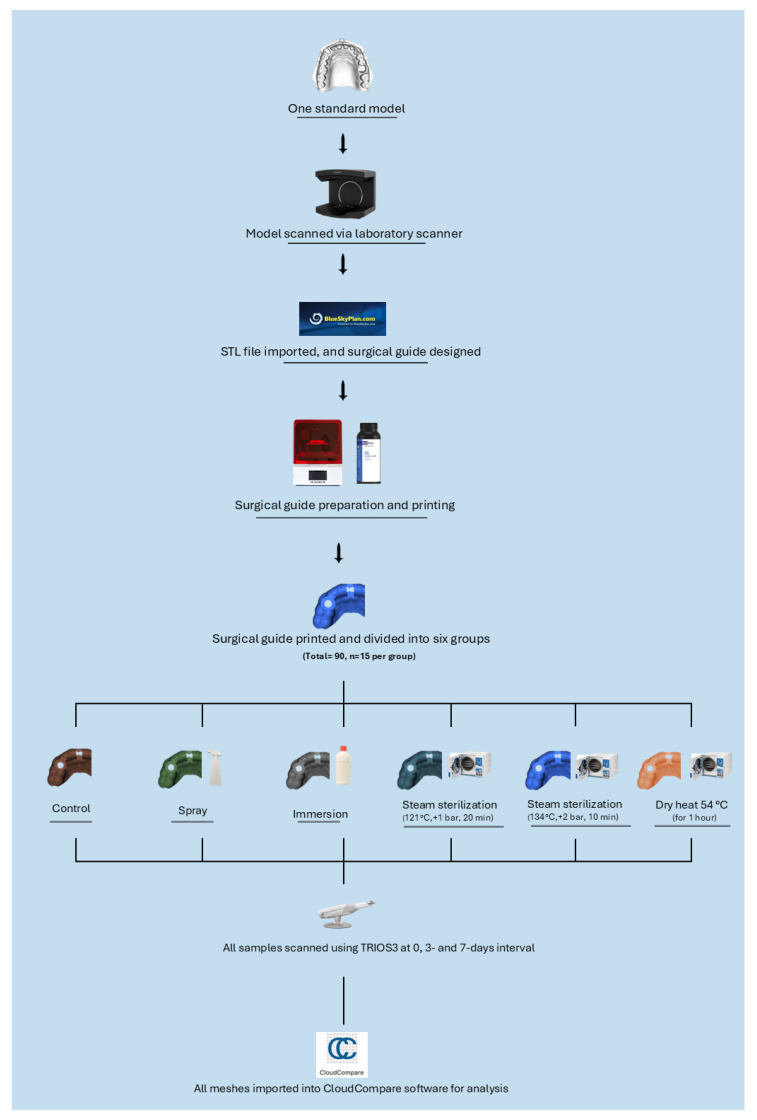
Flowchart of the experiment started by scanning the model using a laboratory scanner. The resulting STL file was imported into BlueSky bio software, where a surgical guide was designed and afterward printed using 3D printing material with an ASIGA UV max printer. The printed surgical guides were then divided into six groups: control, 99% alcohol spray, 99% alcohol immersion with ultrasonication, steam sterilization at 121 °C and 134 °C, and dry heat at −54 °C.

One standardized model (Frasaco, ANA−4 V, Tettnang, Germany) was scanned once using a laboratory scanner (E3;3ShapeA/S). The model’s STL file was imported into Blue Sky Plan (BlueSkyBio, version 4.13.35, New York City, NY, USA). Surgical guides were designed and printed using NextDent surgical guide 3D printing material (NextDent SG, Vertex-Dental B.V., Soesterberg, The Netherlands) with an ASIGA printer (ASIGA Max UV3D light printer, Sydney, Australia). The printed surgical guides were then allocated into six experimental groups for testing under various disinfection protocols (Figure 1).

Group A: Baseline scan of newly printed surgical guide (control group).

Group B: Surgical guide subjected to spray disinfection (using 99% isopropyl alcohol).

Group C: Surgical guide immersed in 99% isopropyl alcohol and ultrasonication for 5 min.

Group D: Surgical guide exposed to dry heat at low temperature 54 °C for 1 h using Sterilizer heating oven (Memmert GmbH, Büchenbach, Germany).

Group E: Surgical guide sterilized using an autoclave cycle (121 °C, +1 bar, for 20 min).

Group F: Surgical guide sterilized using an autoclave cycle (134 °C, +2 bar, for 10 min).

**Figure 2:**
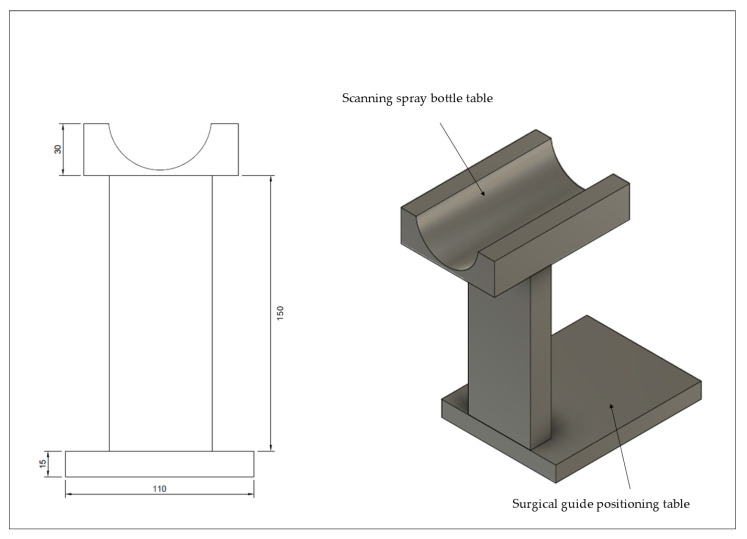
Spraying tool designed and 3D printed, with standard scanning spray distance at 15 cm; scanning spray bottle can be placed in its designated area so that the tip faces downward, directly toward the surgical guide.

Each surgical guide was sprayed once by one experienced operator using a specially designed, 3D-printed spraying device. This spraying device was designed to maintain a fixed distance (15 CM) between the scanning spray tip and the specimen in accordance with the recommendation by Echhpal, Ahmed et al. [12] (Figure 2).

**Figure 3:**
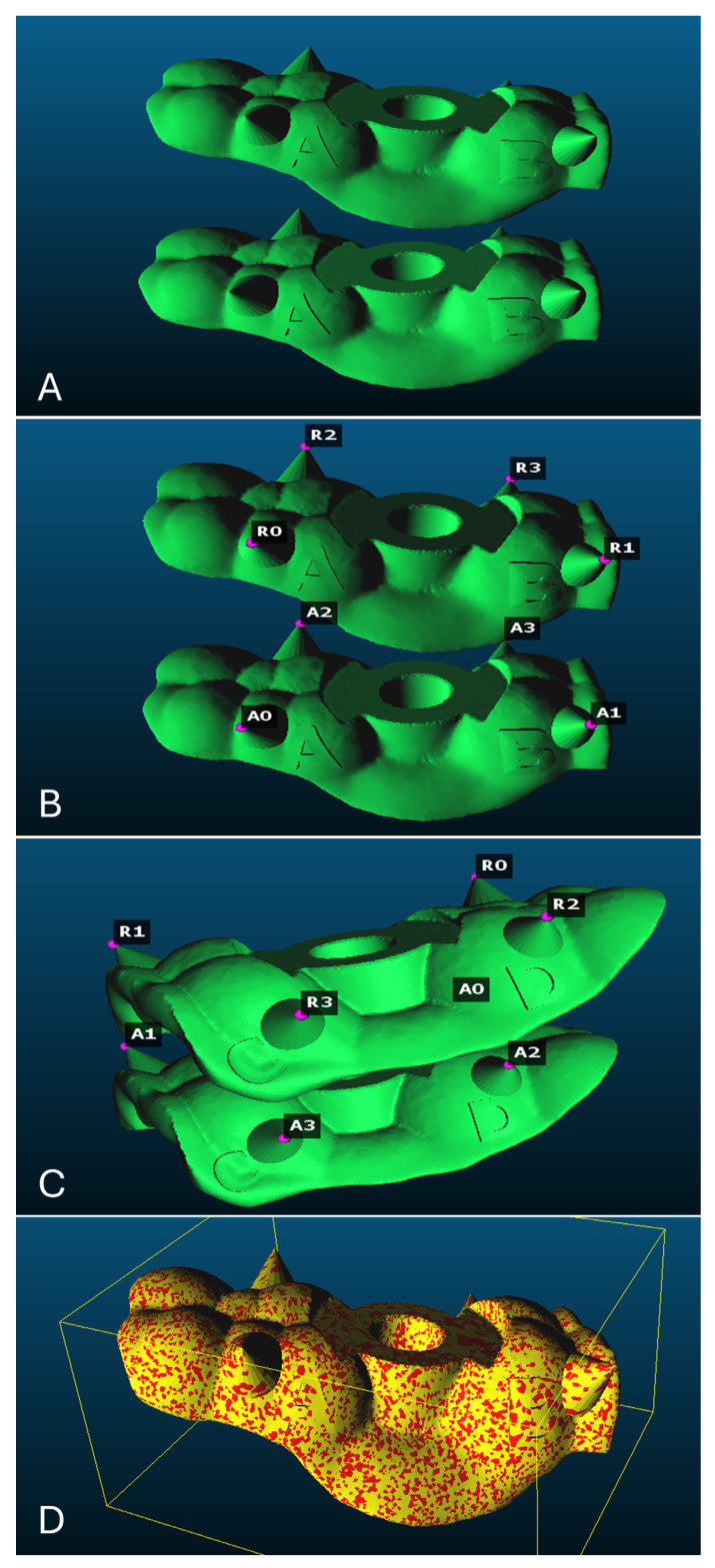
Each mesh contained 4 fixed, pyramid-shaped reference points (**A**,**B**) with four letters to standardize reference points determination sequence; (**C**) the 4 reference points selected on both meshes; (**D**) the two superimposed meshes.

Each group was scanned using a TRIOS 3 intraoral scanner (TRIOS 3; 3Shape A/S). All samples had 4 fixed, pyramid-shaped, clearly visible reference points alongside 4 letters to the standardize reference points determination sequence; scanning was performed following the manufacturer’s scanning strategies at intervals of 0, 3, and 7 days (±3 h, stored in a dry-sealed pouch). Subsequently, the mesh files were imported into CloudCompare software (V2.14 alpha) to assess dimensional stability and evaluate any volumetric changes induced by each protocol on both the fitting and polished surfaces (Figure 3A–D).

A pilot study was conducted to validate the protocol, measure power, and sample size. Partial eta (=0.092, with 80% power and Alpha set to 0.05) was used to calculate the sample size using G*Power software (G*Power, Version 3.1.9.4), revealing that a total of 24 samples were needed. Therefore, we decided to have 90 samples, with 15 samples per group (n = 15).

All data were tabulated and analyzed using the Shapiro–Wilk’s test, Levene’s test, one-way repeated-measures ANOVA, and Tukey’s post hoc test to determine statistical differences (IBM^®^ SPSS^®^ software platform, Ver. 30.0.0).

## 4. Results

A total of 90 samples, divided into six groups (15 samples per group), were subjected to different disinfection treatments and investigated at three time intervals (baseline, three, and seven days). Based on Kolmogorov–Smirnov test results, the data appeared to be normally distributed among all groups except for the Autoclave 121 °C group at baseline, which showed a *p*-value of 0.024 (Table 1).

There was no statistically significant difference in variance between the disinfectant treatments across the different time intervals, indicating that the data were homogeneous (Table 2).

Time had a statistically significant effect on the RMS of scanned surgical guides, indicating that measurements taken at different time intervals differed significantly from one another.

However, the interaction between time and disinfectants was not statistically significant (*p* > 0.05), indicating that the effect of time was consistent across different types of disinfectants (Table 3 and Table 4).

**Chart 1:**
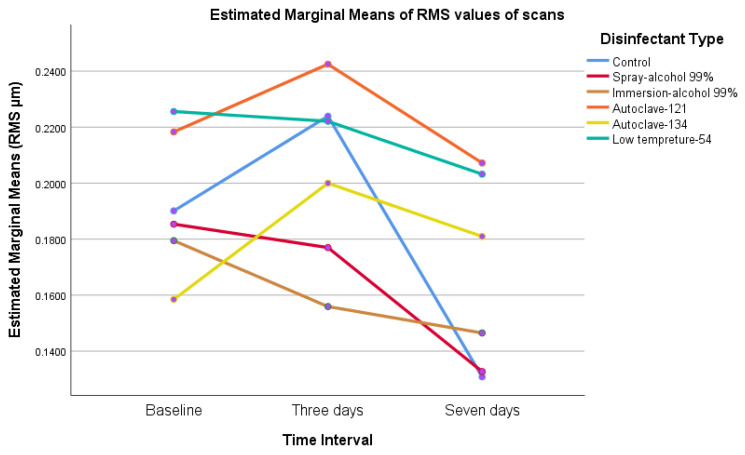
The marginal mean of root mean squared (RMS) values of all groups; we noticed that RMS changes start immediately after printing, then decline by day 7.

**Chart 2:**
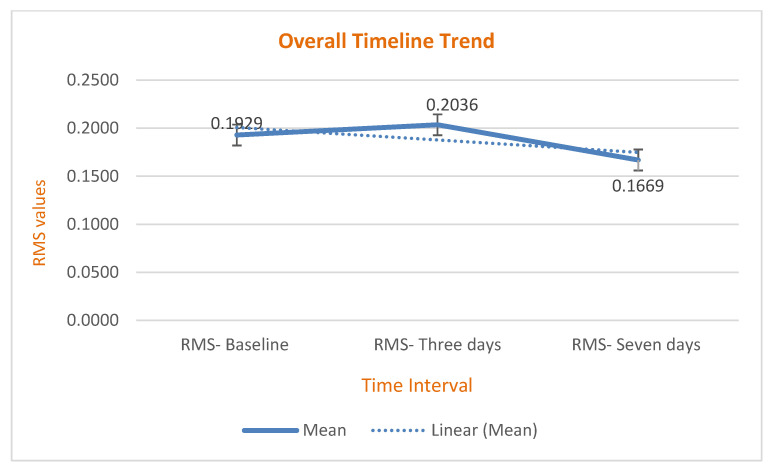
The overall time trend had a specific pattern that started at baseline, increased until day 3, then declined by day 7.

The results showed that the RMS value of scanned surgical guides increased from baseline to day 3, then decreased by day 7 (*p* = 0.00–0.016), indicating that surgical guides became more dimensionally stable seven days after printing and disinfection (Table 5) and (Chart 1 and Chart 2).

**Chart 3:**
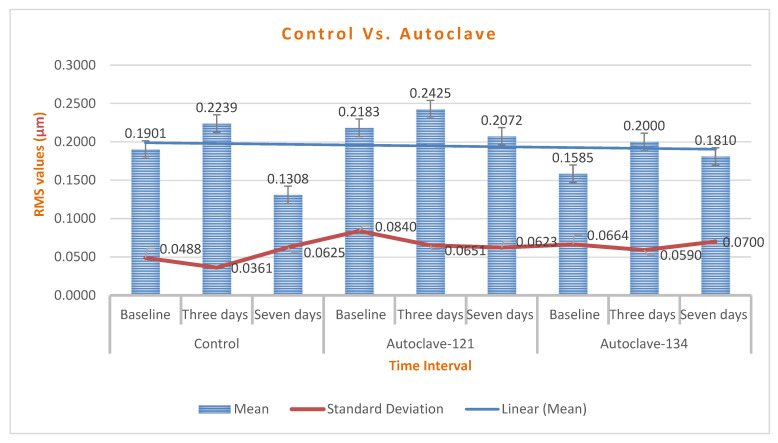
A comparison of RMS changes between control, autoclave 121 °C, and 134 °C groups; the mean of RMS changes was below 1000–2000 µm, which is insignificant.

**Chart 4:**
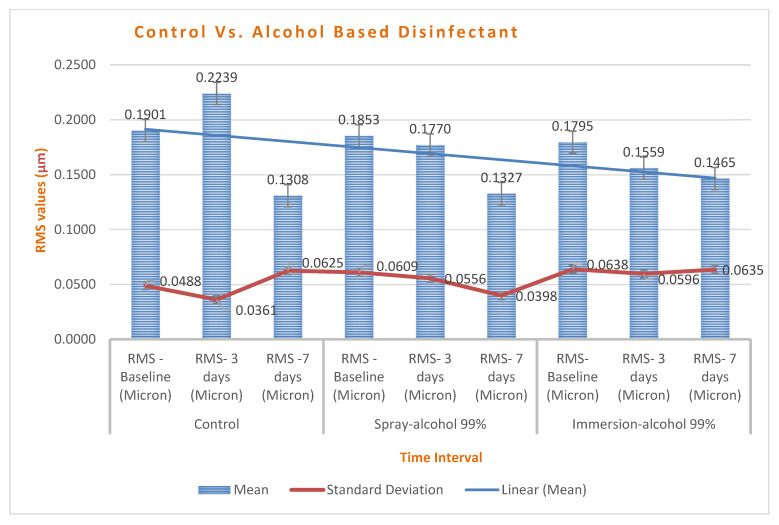
A comparison of RMS changes between control and alcohol-based groups. Spray maintained better stability than immersion, and all groups showed improved stability on day 7.

**Chart 5:**
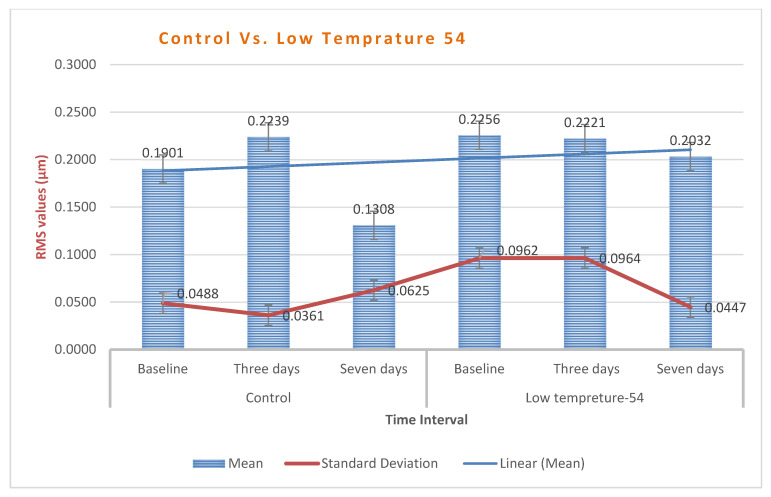
Low-temperature group (54 °C) exhibited higher dimensional changes than the control, yet it is still within an acceptable range.

**Chart 6:**
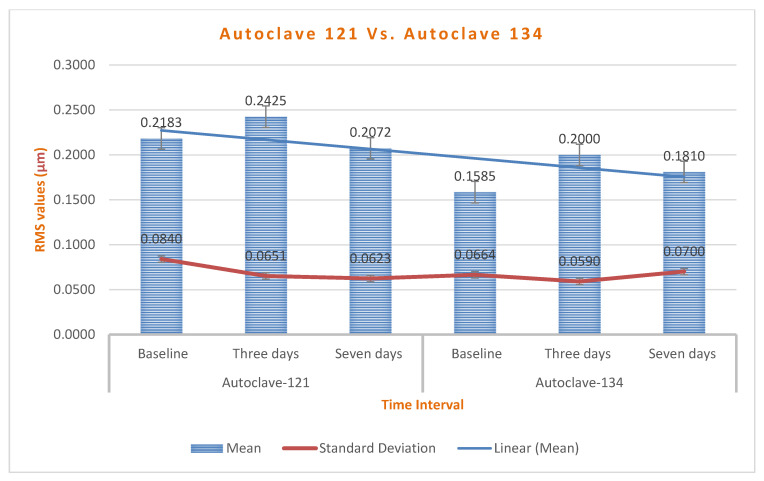
Autoclave 121 °C group revealed superior stability with RMS changes decreasing over time.

Regarding the comparison of disinfectant types, Tukey’s post hoc analysis revealed that RMS changes in the control group were not statistically significant compared to those in all other groups. However, the autoclave 121 °C and low-temperature 54 °C (*p* = 0.002 and 0.001) disinfectant groups showed significantly lower RMS values than both the spray alcohol and immersion alcohol 99% groups (*p* = 0.009 and 0.003, respectively), with values nearly comparable to those of the control group at 7 days (Table 6 and Table 7) (Chart 3, Chart 4, Chart 5 and Chart 6).

Further comparison between the two autoclave groups revealed that the autoclave 121 °C group exhibited significantly lower RMS changes than the autoclave 134 °C group (*p* = 0.045), indicating superior dimensional stability (Table 7) (Chart 6).

## 5. Discussion

This study aims to evaluate the impact of alcohol immersion, alcohol spray, low temperature, and steam sterilization on the dimensional stability of surgical guides, ensuring accurate implant placement and reducing the incidence of implant failure due to infection. The null hypothesis of this study was that there is no statistically significant difference in the dimensional stability of surgical guides after sterilization, low temperature, alcohol spray, and immersion at 3–7 day intervals.

Our study demonstrated that time has a significant impact on the dimensional stability of 3D-printed surgical guides, while the interaction between time and disinfectant is consistent (*p* > 0.05) across all types of sterilization and disinfection. The time trend of RMS changes increases from zero to three days, then declines afterward until day 7, indicating that better dimensional stability is achieved after 7 days.

Similarly, a recent study by Pranno N. et al. observed that deviation from the CAD reference model after printing and sterilization occurred after one month for both control and experimental groups [13]. Ntovaset al. depicted that storage of surgical guides for up to 3 months can affect dimensional accuracy, with RMS for the intaglio surface of the surgical guides ranging from 0.1 to 0.18. To reduce such errors, dark storage media were recommended [14].

Our findings showed that RMS changes in the control group were not statistically significant compared to all other groups. However, the autoclave 121 °C and low-temperature 54 °C disinfectant groups (*p* = 0.002 and 0.001, respectively) showed significantly lower RMS values than both the spray and alcohol 99% immersion groups (*p* = 0.009 and 0.003, respectively), with values nearly comparable to those of the control group at day 7. This aligns with findings by Sharma et al. and Marei et al., who reported no significant effect of steam heat sterilization on the dimensional stability of 3D-printed surgical guides [1,15]. A literature review reported a study by Hüfner et al. that showed minor and significant shrinkage could be observed due to the 3D printing process itself or steam autoclaving cycles, with a need for clinical validation [16]. A study by Russo et al. stated that the stability of 3D-printed surgical guides subjected to autoclave sterilization is limited and clinically insignificant [17]. A recent systematic review reported that implant placement accuracy is considered clinically acceptable when the mean distance deviation falls between 1 and 2 mm and the angular deviation is below 8° [18]. In the same vein, Sharma et al. specified that pre- and post-sterilization RMS values were observed to be below 100 and 200 µm, respectively [1].

With respect to the mechanical properties of 3D-printed surgical guides after sterilization, controversy exists. A study by Pop et al. reported that both 121 °C and 134 °C sterilization cycles alter the mechanical properties of surgical guides [19]. Valls-Esteve et al. concluded in their study that both temperature and exposure time can influence the mechanical behavior of the 3D model. They also recommend that sterilization methods based on materials, technologies, and clinical applications should be followed [20].

Furthermore, a study by Labakoum et al. found that high temperatures can affect the mechanical and geometric properties, and as an alternative, 70% isopropyl alcohol is recommended [21]. On the contrary, a study by Török et al. investigated the effect of plasma sterilization and steam sterilization at 121 °C on the mechanical properties of 3D-printed surgical guides. They found that both methods were suitable for sterilization [22]. A study by Gandarilla et al. found a statistical difference in distortion between chemical (70% isopropyl alcohol) and thermal sterilization (134 °C for 20 min), but it was not clinically significant [23].

Our results showed that 99% alcohol spray and immersion caused greater RMS changes than Autoclave at 121 °C, contradicting previously cited studies. We believe this is mainly due to the imbibition and syneresis phenomena in resin. Upon immersion, the resin material absorbs alcohol or water, causing dimensional expansion. Conversely, evaporation of the absorbed liquid or alcohol leads to volumetric changes resulting in the observed RMS differences. A study by Pfeiffer et al. reported that residual monomers associated with water sorption and solubility could lead to dimensional instability [24]. Additionally, Berli et al. [25] and Perea-Loweryin et al. [26] found that 3D-printed resins exhibited higher water sorption and solubility compared to conventional processed occlusal devices. Regarding various sterilization and disinfection protocols, a study by Li et al. overlaying the guides measured the distance and angle between the cross-shaped marks. They observed significant changes in hydrogen peroxide (H_2_O_2_), glutaraldehyde, autoclaving, and iodophor [27]. In an attempt to investigate the effect of water and saliva absorption by 3D-printed PMMA material, it has been reported that water absorption increases in 3D-printed materials compared to conventional PMMA-based resin [28].

Several studies showed that increasing post-curing time up to 20 min can decrease material solubility and water absorption [29]. Storage media have a markedly greater impact on monomer release, reported to be higher with ethanol than water [30,31,32,33].

Direct and indirect sun exposure can affect the dimensional stability of printed surgical guides. Ntovas et al. [14] and Yousef et al. [34] reported that storage of surgical guides for up to 3 months significantly influences their dimensional stability; therefore, a dark storage environment for guides is recommended. Similarly, Ozden et al. reported that guides stored in a dark and dry medium showed significantly less dimensional changes compared to those stored in wet or light-exposed media, emphasizing the importance of controlling storage media prior to clinical use [35]. An agreement was found with the findings of Pranno et al., as they reported that minimizing unnecessary light exposure is advisable to preserve guide fidelity [13]. However, the existing literature contains studies that contradict. A study reported by Antonopoulou et al. compared occlusal devices stored at dry–light, dry–dark, and wet–dark environments, and the results showed that dry–light environments had the best dimensional stability [36].

Comparing the 121 °C and 134 °C autoclave groups, our results showed that the autoclave at 121 °C exhibited significantly lower RMS changes than at 134 °C (*p* = 0.045), indicating better dimensional stability. This is consistent with Bassiony et al., who found that autoclaving at 121 °C for 15 min resulted in less deformation than at 134 °C, along with total eradication of microorganisms [37].

Preventing medical risks related to surgical site contamination is the main reason that autoclaving is necessary. Studies have shown that immersing acrylic resin surgical guides in disinfectant solutions such as chlorhexidine digluconate, sodium hypochlorite, sodium perborate, or glutaraldehyde does not fully eliminate microbial contamination. Additionally, they confirm that ethanol at concentrations of 70 to 80% is the most effective [7]. This aligns with findings by Sennhenn and colleagues, who stated that 80% alcohol is the most effective disinfectant, achieving complete microbial eradication within 5 min [38]. In contrast, Tallarico et al. reported that 16% of the colony form remained after disinfecting with 70% ethanol for 15 min [6].

Oth et al. investigated low-temperature sterilization methods at 54 °C, especially hydrogen peroxide gas plasma and ethylene oxide gas sterilization, both were effective at this reduced temperature. These methods are well-suited for sterilizing heat-sensitive materials, including 3D-printed templates made from polylactic acid, polyethylene terephthalate glycol, polypropylene, nylon, and both flexible and rigid photopolymer resins [39].

However, gas plasma sterilization was only partially effective, as bacterial growth was detected in two out of five cylinders, suggesting that gas plasma may have limited penetration abilities, especially in sterilizing complex and hollow structures [40].

The biological advantages of autoclaving surgical guides outweigh the potential risks of changes in flexural properties or Vickers hardness. Ultimately, autoclaving remains a clinically relevant and most widely used sterilization method in dental practice [7].

Within the limitations of our study, we recommend that the most effective method to achieve proper sterilization while maintaining the dimensional stability of surgical guides is to autoclave at 121 °C and 1 bar for 20 min, the second most effective method was dry- low temperature of 54 °C for 1 h. Our findings showed that a 99% alcohol spray or immersion protocol can result in high RMS changes after 7 days. Considering our results, we conclude that our null hypothesis was rejected.

## 6. Clinical Implications

Many commonly used 3D printing materials for surgical guides are not currently classified as sterilizable. This lack of standardization presents additional challenges for maintaining effective infection control. Our study showed that 7 days post-printing and sterilization was the most stable phase after autoclaving at 121 °C, +1 bar for 20 min, and a low temperature of 54 °C for 1 h, with minimal RMS changes observed and better dimensional stability of surgical guides.

## 7. Conclusions

The biological benefits of autoclaving surgical guides outweigh the potential risks associated with changes in flexural properties or Vickers hardness. Within the limitations of the present study, the most effective approach for maintaining the dimensional stability of surgical guides is autoclaving at 121 °C, +1 bar for 20 min, and the second most effective technique was dry- low temperature of 54 °C for 1 h. Evidence suggests that the autoclave remains clinically relevant and is the most widely used sterilization method in dental practice.

## 8. Limitations and Future Directions

We did not conduct microbial analysis to correlate which sterilization and disinfection protocol resulted in the total eradication of microorganisms, nor did we analyze mechanical properties; in fact, this is the topic of our upcoming research.

## Figures and Tables

**Table 1:** Tests of normality of samples at different time intervals based on Kolmogorov–Smirnov and Shapiro–Wilk tests.

	Disinfectant Type	Kolmogorov–Smirnov ^a^			Shapiro–Wilk		
		Statistic	df	Sig.	Statistic	df	Sig.
RMS Baseline (μ)	Control	0.168	15	0.200 *	0.959	15	0.684
	Spray-alcohol 99%	0.135	15	0.200 *	0.977	15	0.949
	Immersion-alcohol 99%	0.139	15	0.200 *	0.959	15	0.668
	Autoclave-121	0.236	15	0.024	0.877	15	0.043
	Autoclave-134	0.187	15	0.167	0.923	15	0.210
	Low temperature-54	0.192	15	0.142	0.956	15	0.617
RMS Three days (μ)	Control	0.173	15	0.200 *	0.930	15	0.272
	Spray-alcohol 99%	0.165	15	0.200 *	0.936	15	0.339
	Immersion-alcohol 99%	0.135	15	0.200 *	0.926	15	0.234
	Autoclave-121	0.187	15	0.165	0.919	15	0.183
	Autoclave-134	0.142	15	0.200 *	0.933	15	0.303
	Low temperature-54	0.175	15	0.200 *	0.868	15	0.032
RMS Seven days (μ)	Control	0.117	15	0.200 *	0.963	15	0.739
	Spray-alcohol 99%	0.125	15	0.200 *	0.951	15	0.540
	Immersion-alcohol 99%	0.156	15	0.200 *	0.970	15	0.854
	Autoclave-121	0.154	15	0.200 *	0.901	15	0.100
	Autoclave-134	0.193	15	0.138	0.916	15	0.166
	Low temperature-54	0.121	15	0.200 *	0.950	15	0.521

*. This is a lower bound of the true significance. ^a^. Lilliefors Significance Correction.

**Table 2:** Test of homogeneity of variance at different time intervals.

		Levene Statistic	df1	df2	Sig.
RMS Baseline (μ)	Based on Mean	0.935	5	84	0.463
	Based on Median	0.708	5	84	0.619
	Based on Median and with adjusted df	0.708	5	59.540	0.620
	Based on trimmed mean	0.857	5	84	0.514
RMS Three days (μ)	Based on Mean	1.331	5	84	0.259
	Based on Median	1.210	5	84	0.312
	Based on Median and with adjusted df	1.210	5	56.039	0.317
	Based on trimmed mean	1.347	5	84	0.253
RMS Seven days (μ)	Based on Mean	0.851	5	84	0.518
	Based on Median	0.635	5	84	0.673
	Based on Median and with adjusted df	0.635	5	65.431	0.674
	Based on trimmed mean	0.862	5	84	0.510

**Table 3:** Multivariate tests ^a^ of time effect and time–disinfectant interaction.

Effect		Value	F	Hypothesis df	Error df	Sig.	Partial Eta Squared	Noncent. Parameter	Observed Power ^d^
Time	Pillai’s Trace	0.175	8.824 ^b^	2.000	83.000	0.000	0.175	17.648	0.967
	Wilks’ Lambda	0.825	8.824 ^b^	2.000	83.000	0.000	0.175	17.648	0.967
	Hotelling’s Trace	0.213	8.824 ^b^	2.000	83.000	0.000	0.175	17.648	0.967
	Roy’s Largest Root	0.213	8.824 ^b^	2.000	83.000	0.000	0.175	17.648	0.967
Time * Disinfect	Pillai’s Trace	0.175	1.610	10.000	168.000	0.107	0.087	16.104	0.769
	Wilks’ Lambda	0.832	1.597 ^b^	10.000	166.000	0.111	0.088	15.973	0.764
	Hotelling’s Trace	0.193	1.584	10.000	164.000	0.115	0.088	15.840	0.760
	Roy’s Largest Root	0.125	2.098 ^c^	5.000	84.000	0.074	0.111	10.488	0.668

^a^ Design: Intercept + Disinfect, within-subjects design: time; ^b^ exact statistic; ^c^ the statistic is an upper bound on F that yields a lower bound on the significance level; ^d^ computed using alpha = 0.05.

**Table 4:** Tests of within-subjects contrasts for time effect and time–disinfectant interaction.

Source	Time	Type III Sum of Squares	df	Mean Square	F	Sig.	Partial Eta Squared	Noncent. Parameter	Observed Power ^a^
Time	Linear	0.030	1	0.030	8.178	0.005	0.089	8.178	0.807
	Quadratic	0.034	1	0.034	7.931	0.006	0.086	7.931	0.795
Time * Disinfect	Linear	0.033	5	0.007	1.798	0.122	0.097	8.988	0.590
	Quadratic	0.029	5	0.006	1.365	0.246	0.075	6.824	0.460
Error (Time)	Linear	0.312	84	0.004					
	Quadratic	0.356	84	0.004					

^a^ Computed using alpha = 0.05.

**Table 5:** Pairwise comparisons of different time intervals—baseline, three, and seven days.

(I) Time	(J) Time	Mean Difference (I-J)	Std. Error	Sig. ^b^	95% Confidence Interval for Difference ^b^	
					Lower Bound	Upper Bound
1	2	−0.011	0.010	0.856	−0.035	0.014
	3	0.026 *	0.009	0.016	0.004	0.048
2	1	0.011	0.010	0.856	−0.014	0.035
	3	0.037 *	0.009	0.000	0.014	0.059
3	1	−0.026 *	0.009	0.016	−0.048	−0.004
	2	−0.037 *	0.009	0.000	−0.059	−0.014

Based on estimated marginal means. *. The mean difference is significant at the 0.05 level. ^b^ Adjustment for multiple comparisons: Bonferroni.

**Table 6:** Pairwise comparisons of various disinfectant treatment protocols.

(I) Disinfectant Type	(J) Disinfectant Type	Mean Difference (I-J)	Std. Error	Sig. ^b^	95% Confidence Interval for Difference ^b^	
					Lower Bound	Upper Bound
Control	Spray-alcohol 99%	0.017	0.015	1.000	−0.027	0.060
	Immersion-alcohol 99%	0.021	0.015	1.000	−0.023	0.065
	Autoclave-121	−0.041	0.015	0.087	−0.085	0.003
	Autoclave-134	0.002	0.015	1.000	−0.042	0.046
	Low temperature-54	−0.035	0.015	0.254	−0.079	0.008
Spray-alcohol 99%	Control	−0.017	0.015	1.000	−0.060	0.027
	Immersion-alcohol 99%	0.004	0.015	1.000	−0.039	0.048
	Autoclave-121	−0.058 *	0.015	0.002	−0.101	−0.014
	Autoclave-134	−0.015	0.015	1.000	−0.059	0.029
	Low temperature-54	−0.052 *	0.015	0.009	−0.096	−0.008
Immersion-alcohol 99%	Control	−0.021	0.015	1.000	−0.065	0.023
	Spray-alcohol 99%	−0.004	0.015	1.000	−0.048	0.039
	Autoclave-121	−0.062 *	0.015	0.001	−0.106	−0.018
	Autoclave-134	−0.019	0.015	1.000	−0.063	0.025
	Low temperature-54	−0.056 *	0.015	0.003	−0.100	−0.013
Autoclave-121	Control	0.041	0.015	0.087	−0.003	0.085
	Spray-alcohol 99%	0.058 *	0.015	0.002	0.014	0.101
	Immersion-alcohol 99%	0.062 *	0.015	0.001	0.018	0.106
	Autoclave-134	0.043	0.015	0.061	−0.001	0.087
	Low temperature-54	0.006	0.015	1.000	−0.038	0.050
Autoclave-134	Control	−0.002	0.015	1.000	−0.046	0.042
	Spray-alcohol 99%	0.015	0.015	1.000	−0.029	0.059
	Immersion-alcohol 99%	0.019	0.015	1.000	−0.025	0.063
	Autoclave-121	−0.043	0.015	0.061	−0.087	0.001
	Low temperature-54	−0.037	0.015	0.183	−0.081	0.007
Low temperature-54	Control	0.035	0.015	0.254	−0.008	0.079
	Spray-alcohol 99%	0.052 *	0.015	0.009	0.008	0.096
	Immersion-alcohol 99%	0.056 *	0.015	0.003	0.013	0.100
	Autoclave-121	−0.006	0.015	10.000	−0.050	0.038
	Autoclave-134	0.037	0.015	0.183	−0.007	0.081

Based on estimated marginal means. *. The mean difference is significant at the 0.05 level. ^b^. Adjustment for multiple comparisons: Bonferroni.

**Table 7:** Multiple comparisons of various disinfectant treatment protocols.

	(I) Disinfectant Type	(J) Disinfectant Type	Mean Difference (I-J)	Std. Error	Sig.	95% Confidence Interval	
						Lower Bound	Upper Bound
Tukey HSD	Control	Spray-alcohol 99%	0.016607	0.0145053	0.861	−0.025699	0.058912
		Immersion-alcohol 99%	0.020996	0.0145053	0.698	−0.021310	0.063301
		Autoclave-121	−0.041042	0.0145053	0.063	−0.083348	0.001263
		Autoclave-134	0.001813	0.0145053	1.000	−0.040492	0.044119
		Low temperature-54	−0.035336	0.0145053	0.156	−0.077641	0.006970
	Spray-alcohol 99%	Control	−0.016607	0.0145053	0.861	−0.058912	0.025699
		Immersion-alcohol 99%	0.004389	0.0145053	1.000	−0.037916	0.046694
		Autoclave-121	−0.057649 *	0.0145053	0.002	−0.099954	−0.015344
		Autoclave-134	−0.014793	0.0145053	0.910	−0.057099	0.027512
		Low temperature-54	−0.051942 *	0.0145053	0.007	−0.094248	−0.009637
	Immersion-alcohol 99%	Control	−0.020996	0.0145053	0.698	−0.063301	0.021310
		Spray-alcohol 99%	−0.004389	0.0145053	1.000	−0.046694	0.037916
		Autoclave-121	−0.062038 *	0.0145053	0.001	−0.104343	−0.019732
		Autoclave-134	−0.019182	0.0145053	0.772	−0.061488	0.023123
		Low temperature-54	−0.056331 *	0.0145053	0.003	−0.098636	−0.014026
	Autoclave-121	Control	0.041042	0.0145053	0.063	−0.001263	0.083348
		Spray-alcohol 99%	0.057649 *	0.0145053	0.002	0.015344	0.099954
		Immersion-alcohol 99%	0.062038 *	0.0145053	0.001	0.019732	0.104343
		Autoclave-134	0.042856 *	0.0145053	0.045	0.000550	0.085161
		Low temperature-54	0.005707	0.0145053	0.999	−0.036599	0.048012
	Autoclave-134	Control	−0.001813	0.0145053	1.000	−0.044119	0.040492
		Spray-alcohol 99%	0.014793	0.0145053	0.910	−0.027512	0.057099
		Immersion-alcohol 99%	0.019182	0.0145053	0.772	−0.023123	0.061488
		Autoclave-121	−0.042856 *	0.0145053	0.045	−0.085161	−0.000550
		Low temperature-54	−0.037149	0.0145053	0.119	−0.079454	0.005156
	Low temperature-54	Control	0.035336	0.0145053	0.156	−0.006970	0.077641
		Spray-alcohol 99%	0.051942 *	0.0145053	0.007	0.009637	0.094248
		Immersion-alcohol 99%	0.056331 *	0.0145053	0.003	0.014026	0.098636
		Autoclave-121	−0.005707	0.0145053	0.999	−0.048012	0.036599
		Autoclave-134	0.037149	0.0145053	0.119	−0.005156	0.079454
Bonferroni	Control	Spray-alcohol 99%	0.016607	0.0145053	1.000	−0.027221	0.060434
		Immersion-alcohol 99%	0.020996	0.0145053	1.000	−0.022832	0.064823
		Autoclave-121	−0.041042	0.0145053	0.087	−0.084869	0.002785
		Autoclave-134	0.001813	0.0145053	1.000	−0.042014	0.045641
		Low temperature-54	−0.035336	0.0145053	0.254	−0.079163	0.008492
	Spray-alcohol 99%	Control	−0.016607	0.0145053	1.000	−0.060434	0.027221
		Immersion-alcohol 99%	0.004389	0.0145053	1.000	−0.039438	0.048216
		Autoclave-121	−0.057649 *	0.0145053	0.002	−0.101476	−0.013822
		Autoclave-134	−0.014793	0.0145053	1.000	−0.058621	0.029034
		Low temperature-54	−0.051942 *	0.0145053	0.009	−0.095769	−0.008115
	Immersion-alcohol 99%	Control	−0.020996	0.0145053	1.000	−0.064823	0.022832
		Spray-alcohol 99%	−0.004389	0.0145053	1.000	−0.048216	0.039438
		Autoclave-121	−0.062038 *	0.0145053	0.001	−0.105865	−0.018211
		Autoclave-134	−0.019182	0.0145053	1.000	−0.063009	0.024645
		Low temperature-54	−0.056331 *	0.0145053	0.003	−0.100158	−0.012504
	Autoclave-121	Control	0.041042	0.0145053	0.087	−0.002785	0.084869
		Spray-alcohol 99%	0.057649 *	0.0145053	0.002	0.013822	0.101476
		Immersion-alcohol 99%	0.062038 *	0.0145053	0.001	0.018211	0.105865
		Autoclave-134	0.042856	0.0145053	0.061	−0.000972	0.086683
		Low temperature-54	0.005707	0.0145053	1.000	−0.038121	0.049534
	Autoclave-134	Control	−0.001813	0.0145053	1.000	−0.045641	0.042014
		Spray-alcohol 99%	0.014793	0.0145053	1.000	−0.029034	0.058621
		Immersion-alcohol 99%	0.019182	0.0145053	1.000	−0.024645	0.063009
		Autoclave-121	−0.042856	0.0145053	0.061	−0.086683	0.000972
		Low temperature-54	−0.037149	0.0145053	0.183	−0.080976	0.006678
	Low temperature-54	Control	0.035336	0.0145053	0.254	−0.008492	0.079163
		Spray-alcohol 99%	0.051942 *	0.0145053	0.009	0.008115	0.095769
		Immersion-alcohol 99%	0.056331 *	0.0145053	0.003	0.012504	0.100158
		Autoclave-121	−0.005707	0.0145053	1.000	−0.049534	0.038121
		Autoclave-134	0.037149	0.0145053	0.183	−0.006678	0.080976

Based on observed means. The error term is mean squared (Error) = 0.002. *. The mean difference is significant at the 0.05 level.

## Data Availability

The original contributions presented in this study are included in the article. Further inquiries can be directed to the corresponding author.
